# Tracking tumor evolution during the first-line treatment in brain glioma *via* serial profiling of cell-free tumor DNA from tumor *in situ* fluid

**DOI:** 10.3389/fonc.2023.1238607

**Published:** 2023-10-17

**Authors:** Zhiyuan Sheng, Chaojie Bu, Jie Mei, Sensen Xu, Ziyue Zhang, Guangzhong Guo, Yushuai Gao, Liyuan Xing, Zhongcan Chen, Juha Hernesniemi, Ajmal Zemmar, Xingyao Bu

**Affiliations:** ^1^ Department of Neurosurgery, Zhengzhou University People’s Hospital, Henan Provincial People’s Hospital, Zhengzhou, China; ^2^ Juha International Center for Neurosurgery, Henan Provincial People’s Hospital, Zhengzhou, China; ^3^ Department of Psychological Medicine, Henan Provincial People’s Hospital, Zhengzhou, China; ^4^ Department of Nursing, Henan Provincial People’s Hospital, Zhengzhou, China

**Keywords:** glioma, cell-free tumor DNA, tumor evolution, tumor *in situ* fluid, precision medicine

## Abstract

**Objective:**

Tumor *in situ* fluid (TISF) refers to the fluid within surgical cavities of glioma. Several studies preliminarily proved the value of cell-free tumor DNA (cf-tDNA) from TISF in the dynamic characterization of the glioma genome. Here, we assessed the potential utility of TISF cf-tDNA in broad aspects of tumor evolution under therapeutic pressure.

**Methods:**

This study was conducted under an Institutional Review Board-approved protocol at Henan Provincial People’s Hospital (China). Cf-tDNA samples were sequenced with a designed 68-gene panel. A total of 205 cf-tDNA samples from 107 patients were studied. The clinical relevance of serial cf-tDNA profiling during the postoperative course was analyzed.

**Results:**

At least one tumor mutations were detected in 179/205 (87.3%) TISF cf-tDNA samples. Serial cf-tDNA was complementary to molecular residual disease and to initial tumors. Serial cf-tDNA revealed the selection of pre-existing mismatch repair-deficient cells by temozolomide as a resistant mechanism. Cf-tDNA parameters during treatment were predictive of recurrence, and serial cf-tDNA monitoring had diagnostic value for early recurrence. A total of 223 potentially actionable genomic alterations were assessed in cf-tDNA samples, wherein 78% were not found in any tumor tissue.

**Conclusions:**

In conclusion, serial TISF cf-tDNA profiling is valuable in tracking the tumor evolution of glioma during treatment and may be a feasible non-invasive option for monitoring glioma in future prospective studies and clinical practice.

## Introduction

Trajectories of genomic alterations in gliomas are highly variable and patient-specific (1). At recurrence, the tumor genome is quite different from that of the initial tumor from the same patient (1–3). In addition, there is little known about the *in vivo* evolution of the glioma genome during the treatment, where pre- and in-treatment biopsies are required. However, in the real world, it is not uncommon that repeated tumor tissue biopsies are not available (4). These challenges provide substantial motivation to better understand genomic evolution and mechanisms of resistance to inform the development of new therapeutic strategies for this brain malignancy.

Multiple cancer practitioners are working on incorporating cell-free tumor DNA (cf-tDNA) profiling into clinical practice to aid in disease surveillance, characterize tumor heterogeneity, define mechanisms of resistance, and direct stratified treatment (5–7). To date, most research in cf-tDNA has focused on blood-based biomarkers (4). However, cf-tDNA can also be obtained from various non-blood sources, such as urine, pleural or peritoneal fluid, and cerebrospinal fluid (CSF). These non-blood sources might offer unique advantages for tumors at special locations over plasma (8). For gliomas, it has been demonstrated that CSF cf-tDNA is more enriched and valuable compared to plasma (9–11). Meantime, some concerns about CSF cf-tDNA for gliomas are raised as well. Owing to the invasion nature of lumbar puncture, obtaining samples for translational research might be ethically challenging (8, 10). In addition, the sensitivity of CSF cf-tDNA is not uniformly high and is associated with tumor progression, tumor burden, and tumor location (9, 11).

We previously found that the local fluid within surgical cavities of gliomas was a novel source of glioma-derived cf-tDNA. We called it tumor *in situ* fluid (TISF) to distinguish it from the global CSF in the ventricular system and subarachnoid space (12). During the postoperative course, TISF can be repeatedly collected non-invasively through an intraoperatively implanted reservoir that connects the surgical cavity and the subscalp (12). As early studies suggest sampling closest to the tumor may increase sensitivity (10), we indeed found that TISF cf-tDNA is sensitive to low tumor burden, which allows *de novo* monitoring of gliomas after resection surgery (13–15). Thus, utilizing serial TISF cf-tDNA profiling may conduce to the understanding of glioma genomic evolution during treatment and has potential for monitoring, early relapse diagnosis, and therapeutic decision-making implications for patients with this lethal brain cancer.

## Methods

### Patients and sample collection

This study enrolled patients with glioma undergoing treatment at Henan Provincial People’s Hospital in China between July 2016 and December 2021. These patients received intraoperative implantation of a reservoir into the surgical cavity as the reservation for postoperative diagnosis and treatment. In principle, patients received surgical resection, chemoradiotherapy for high-grade gliomas, and adjuvant temozolomide (TMZ) chemotherapy until relapse; after recurrence, patients would undergo a second surgery or receive bevacizumab (BEV), anti-programmed cell death-1 (anti-PD-1), or BEV plus anti-PD-1 combined therapy. Serial TISF samples were collected for cf-tDNA sequencing during the postoperative standard of care and were subsequently retrospectively analyzed. Blood samples for germline DNA controls from each patient were also obtained. This study was approved by the local institutional review board, and all patients or their next of kin provided written informed consent for any procedure. Samples from 116 patients were evaluated for analysis, wherein 107 patients had paired initial tumor tissue and cf-tDNA samples sequenced with a targeted panel.

### DNA extraction

EDTA tubes containing whole blood samples or *in situ* TISF were centrifuged for 10 min at 1,900 *g*, and the supernatants from these samples were further centrifuged for 10 min at 16,000 *g*. Samples were then collected and stored at − 80°C until extraction. Genomic DNA was extracted from fresh tumor tissue using the QIAamp DNA Tissue & Blood Kit (Qiagen, Germantown, MD, USA). Cell-free DNA was extracted from plasma and TISF using the MagMAX™ CellFree DNA Isolation Kit (Thermo Fisher Scientific, Waltham, MA, USA). Finally, all isolated DNAs were quantified with the Qubit 2.0 Fluorometer using the Qubit dsDNA HS Assay kit (Life Technologies, Carlsbad, CA, USA) according to the recommended protocol.

### Next-generation sequencing library preparation and sequencing data analysis

Genomic DNA was sheared into 150–200- bp fragments using the Covaris M220 Focused-ultrasonicator™ Instrument (Covaris, Woburn, MA, USA). Fragmented DNA and cf-tDNA libraries were constructed using the KAPA HTP Library Preparation Kit (Illumina platforms; KAPA Biosystems, Wilmington, MA, USA) following the manufacturer’s instructions. DNA libraries were captured using a designed brain tumor panel of 68 genes (Genetron Health, Beijing, China) that included major brain tumor-related genes. The captured samples were subjected to Illumina HiSeq X-Ten for paired-end sequencing.

Sequencing reads from the HiSeq X-Ten platform were demultiplexed, allowing zero mismatches in barcodes, and the read quality statistics were calculated by FastQC.

Sequence adapters and low-quality regions were removed with Trimmomatic (v0.36) and then mapped to the hg19 reference genome with BWA (v0.7.10). PCR duplicates were marked using Picard. Local realignment was run using GATK. Pileup files that were converted from bam files were generated for the genomic regions targeted by exome enrichment. With the use of the pileup file as input, single-nucleotide variant (SNV) or indel was identified using SAMtools (v0.1.1722) and Pindel; structural variation was detected using Crest. The criteria we adopted for retaining a mutation in TISF and plasma cf-tDNA were that it had an allele fraction of ≥ 0.1% and a total of ≥ 4 reads. Additionally, known recurrent loci were manually reviewed using Integrative Genomics Viewer (IGV v2.3.34) in the target tissue as compared to the normal blood DNA. All mutations were annotated for genes and function as well as repeated genomic regions using ANNOVAR, Oncotator, and Vep. The dbNSFP and the Exome Aggregation Consortium (ExAC) database were used to filter out either the benign mutations with pp2_hdiv score < 0.452 or polymorphic non-synonymous mutation sites. Finally, all mutations were annotated for genes using ANNOVAR, Oncotator, and Vep.

### Definition of concordance between tumor tissues and paired cf-tDNA samples

Three terms, tissue dominant (TD), tissue complementary (TC), and basically consistent (BC), were defined to describe the degree of consistency between a tumor tissue and the paired cf-tDNA sample. Taking the initial tumor tissue as the baseline comparison, a paired cf-tDNA sample was described TD if it was negative, harbored no dominant subclonal mutation, or only had partial tissue-shared mutations; TC if it had at least one dominant subclonal mutation that was not shared with tissue; or BC if all mutations in the tissue were detected as the whole dominant subclonal mutations in the cf-tDNA sample. If a cf-tDNA mutation had variant allele fraction (VAF) > 0.6 before postoperative antitumor treatment, VAF > 0.8 during adjuvant treatment, or VAF ≥ 1.0 at recurrence, it was considered a dominant mutation in the cf-tDNA sample.

### Statistical analysis

All statistical calculations were performed using Prism 9.4.1 for MacOS. Wilcoxon matched-pairs signed rank test was used for the comparison of maximal VAF or number of mutations (NOM) in paired tissue and cf-tDNA samples. The Mann–Whitney test was used for the comparison of VAF in non-paired tissue and cf-tDNA samples. Chi-square or Fisher’s exact test was used for the comparison of contingency distribution or correlation between groups. Progression-free survival (PFS) analysis used a log-rank test. *p* < 0.05 was considered significant. All *p*- values were two-sided.

## Results

### A majority of TISF cf-tDNA samples have tumor-derived genomic alterations

**Figure 1:**
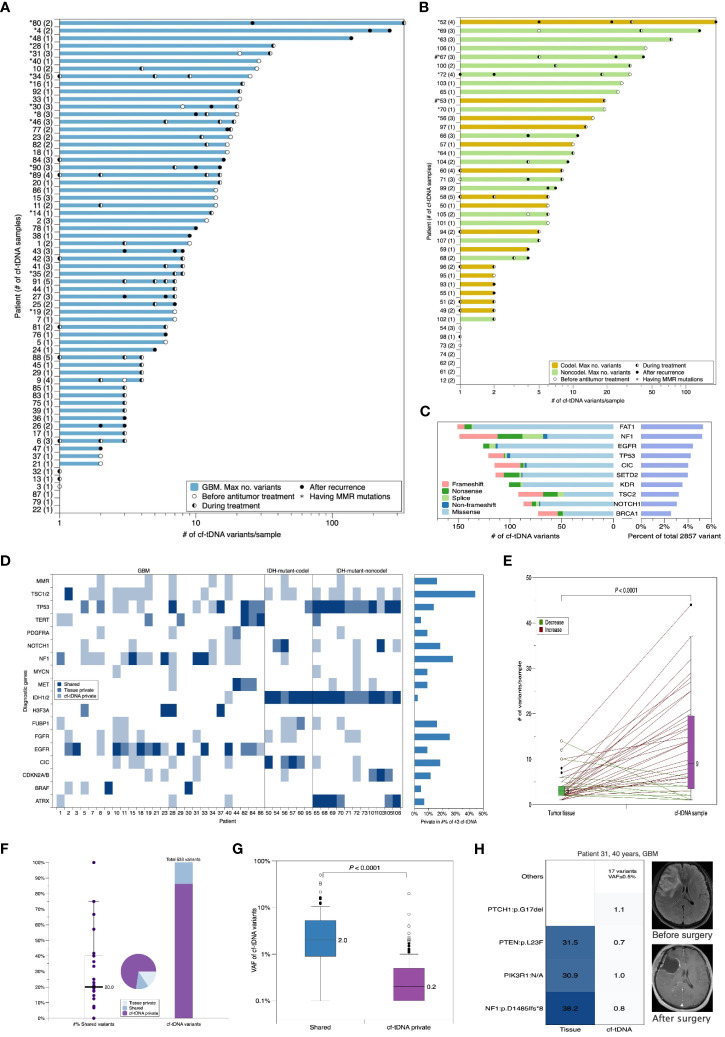
Cf-tDNA is prevalent in TISF, and baseline cf-tDNA represents molecular residual disease of glioma. **(A, B)** Summary of number of genetic variants identified per cf-tDNA sample for each patient. The maximal cf-tDNA variants for each patient are shown with each cf-tDNA sample variant number indicated by a bar. The number of cf-tDNA samples per patient is indicated in parentheses on the y-axis. Patients with cf-tDNA (*) or tumor tissues (#) having mismatch repair (MMR) mutations (MLH1, MSH2, MSH6, and PMS2) are indicated. **(C)** The top 10 most prevalent mutated genes in all cf-tDNA samples. **(D)** Oncoplot of diagnostic genes and MMR mutations in temporally paired tumor tissues and cf-tDNA samples from 43 evaluable patients. These shown genes were referred from the WHO CNS5. **(E)** Comparison of number of mutations in paired tumor and cf-tDNA samples. Wilcoxon matched-pairs signed rank test. **(F)** Fraction of shared versus private mutations in cf-tDNA samples. **(G)** VAF of mutations by those identified in cf-tDNA only versus those found in both tumor and cf-tDNA. **(H)** Illustrative example for cf-tDNA complements tumor genomic landscapes. cf-tDNA, cell-free tumor DNA; TISF, tumor *in situ* fluid; VAF, variant allele fraction.

Serial cf-tDNA analysis was performed on TISF of 107 evaluable patients with glioma that had paired tumor tissues, including the three major subtypes: IDH mutant without co-deletion of chromosome 1p/19q (IDH-mutant-noncodel; n = 25), IDH mutant and chromosome 1p/19q co-deleted (IDH-mutant-codel; n = 17), and IDH wild type (glioblastoma (GBM); n = 65). The median age at the time of the first TISF cf-tDNA sample was 51 years, and a total of 205 cf-tDNA samples were sequenced (median of 2 and range 1–5 cf-tDNA samples/patient). At least one tumor-derived genomic alteration was found in 179/205 (87.3%) of samples (median of 5 cf-tDNA variants/sample; range 0–336 variants/sample). The 26 (12.7%) negative cf-tDNA samples were all collected after the initiation of postoperative radio- or chemotherapy. A total of 2,857 genomic mutations were detected in the cf-tDNA samples, and the most frequently mutated genes were FAT1 (151/2,857, 5.3%), NF1 (148/2,857, 5.2%), and TP53 (137/2,857, 4.8%) (**Table 1**; **Figures 1A–C**).

**Table 1:** Patient demographics and TISF cf-tDNA characteristics.

Characteristic	Total patients (N = 107)
Gender	
Male, no (%)	50 (46.7)
Female, no (%)	57 (53.3)
Median age at diagnosis, years (range)	51 (8–80)
Diagnosis	
IDH wild type, no (%)	65 (60.7)
IDH-mutant-noncodel, no (%)	25 (23.4)
IDH-mutant-codel, no (%)	17 (15.9)
WHO grade	
2, no (%)	15 (14.0)
3, no (%)	16 (15.0)
4, no (%)	76 (71.0)
Postoperative first-line therapy	
RCT + TMZ, no (%)	78 (72.9)
TMZ only, no (%)	29 (27.1)
**Later-line therapy post-relapse**	81
Re-resection, no (%)	8 (9.8)
BEV, no (%)	46 (56.8)
Anti-PD-1, no (%)	14 (17.3)
P + B, no (%)	21 (25.9)
No. of patients cf-tDNA at disease stage	
Before antitumor therapy, no (%)	43 (40.2)
During adjuvant treatment, no (%)	71 (66.4)
At recurrence, no (%)	29 (27.1)
Other stages, no (%)	26 (24.3)
**No. of cf-tDNA samples per patient— total**	205
1 per patient, no (%)	51 (47.7)
2 per patient, no (%)	27 (25.2)
3 per patient, no (%)	20 (18.7)
4 per patient, no (%)	5(4.7)
5 per patient, no (%)	4 (3.7)
**No. of mutations per cf-tDNA sample**, **median (range)**	5 (0-336)
0 (negative), no (%)	26 (12.7)
1~10, no (%)	123 (60.0)
>10, no (%)	56 (27.3)

TISF, tumor in situ fluid; cf-tDNA, cell-free tumor DNA; TMZ, temozolomide; BEV, bevacizumab; RCT, radio-chemotherapy.

### Cf-tDNA before antitumor treatment represents molecular residual disease of gliomas

A total of 43 patients had evaluable cf-tDNA samples before the initiation of antitumor treatment after the initial surgery. All patients had tumor-derived mutations found in their liquid samples. First, we compared diagnostic genetic alterations (16) between tumor tissues and temporally paired cf-tDNA samples (within 45 days). We found that 14.0% (6/43) of patients’ cf-tDNA samples and paired tissues shared the same diagnostic alterations, 18.6% (8/43) cf-tDNA samples contained none or a part of tissue-shared diagnostic variants, and the remaining 67.4% (29/43) cf-tDNA samples were detected to have at least one diagnostic mutation that was not found in paired tissues (**Figure 1D**). The most frequent TISF-private diagnostic mutations were TSC1/2 (in 19/43 patients), NF1 (in 12/43 patients), and FGFR1/3 (in 11/43 patients) (**Figure 1D**). The three genetic alterations were commonly related to the diagnosis of low-grade or circumscribed astrocytic gliomas (16). Considering general alterations, cf-tDNA samples contained far more mutations than paired tissues (median 9 vs 3 mutations/sample, *p* < 0.0001, Wilcoxon matched-pairs signed rank test) (**Figure 1E**), and 86.3% of them (460/533) were private; the median percentage of tissue-shared mutations was only 20% (0%–100%) (**Figure 1F**).

Volume decompression caused by surgical resection resulted in a very low proportion of cf-tDNA to cell-free DNA in TISF. Therefore, the VAFs of cf-tDNA mutations were expectedly low (median VAF 0.2%, range 0.1%–49.9%); among them, the higher VAFs were mainly concentrated on tissue-shared mutations (73 shared vs 460 cf-tDNA-private, median VAF 2.0% vs 0.2%, *p* < 0.0001, Mann–Whitney test) (**Figure 1G**). However, some cf-tDNA-private mutations also had higher VAFs, which might indicate that the residual disease had a genomic structure that was not completely covered by tissue sequencing. For example, patient 31 is a 40-year-old man with GBM, and we detected three mutations— NF1:p.D1485lfs*8, PIK3R1:N/A, and PTEN:p.L23F— in his tumor tissue. The paired cf-tDNA sample was detected to have 21 mutations, including the three shared mutations with VAFs of 0.8%, 1.0%, and 0.7% and 18 private mutations, wherein 17 had VAF of less than 0.5%, except for PTCH1:p.G17del, which had a higher VAF at 1.1%. The eccentrically high mutational abundance of the private PTCH1:p.G17del may represent the supplementary role of TISF cf-tDNA for tumor tissues (**Figure 1H**).

We failed to find the NOM or the maximal VAF (mVAF) in the cf-tDNA samples correlated with MRI-measured residual disease, glioma subtypes, or other features of gliomas (**Supplementary Table 3**), suggesting that shedding of tumor DNA into TISF may be determined by other non-volume characteristics of glioma, or TISF cf-tDNA is supplementary information not just a substitute for the tumor itself.

Collectively, the abundant mutations in the TISF cf-tDNA reflected the molecular residual disease and complemented the genomic heterogeneity of tumor tissues.

### Dynamic consistency between paired cf-tDNA and tumor tissue genomes

**Figure 2:**
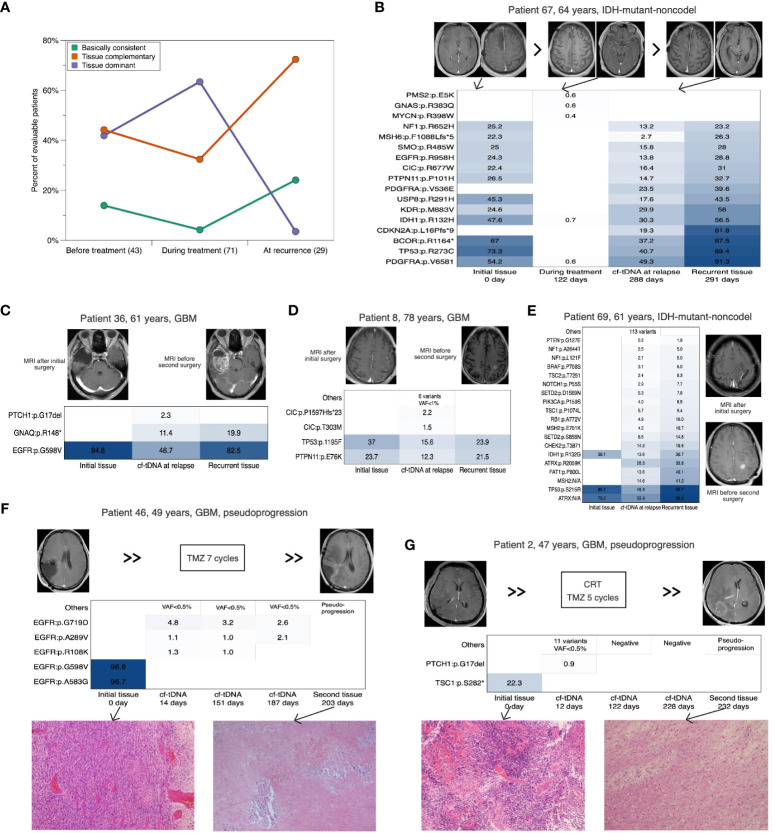
Dynamic consistency between paired cf-tDNA and tumor genomes. **(A)** Horizontal comparison of the consistency at three timepoints. **(B)** Cf-tDNA dynamics in patient 67 at different timepoints. **(B–E)** Comparison between initial tumor, cf-tDNA at recurrence, and recurrent tumor in four evaluable glioma patients. **(F, G)** Serial cf-tDNA profiling in two patients identified as pseudo-progression. cf-tDNA, cell-free tumor DNA.

We analyzed the dynamic consistency of tumor genomes between initial tissues and the paired cf-tDNA samples of three representative timepoints (before antitumor therapy, during chemotherapy, and at recurrence) after surgery. Before antitumor therapy, among 43 evaluable patients, the percentages of TD and TC cf-tDNA samples were similar (41.9% vs 44.2%), and the remaining six (13.9%) were BC samples. During the TMZ chemotherapy, the proportion of TD cf-tDNA samples was twice that of TC samples (63.4% vs 32.4%) in 71 evaluable patients, and only three (4.2%) were BC. When it came to recurrence, of 29 evaluable cf-tDNA samples, 21 (72.4%) were TC and seven (24.1%) were BC, and only one (3.5%) sample was TD (**Figure 2A**). The longitudinal analysis of 15 patients’ cf-tDNA samples showed a similar developing trend. That is, most patients’ cf-tDNA samples evolved from TD during the chemotherapy into TC at recurrence. One example was with patient 67, a 64-year-old man with IDH-mutant-noncodel glioma; after the second period of adjuvant TMZ treatment, five low-VAF (<0.8%) variants were detected in his first cf-tDNA sample without imaging findings, while 5 months later, 24 mutations with elevated VAFs (the maximal 49.3%) emerged in the relapsed cf-tDNA sample, wherein some were not found in the initial tumor tissue (**Figure 2B**).

In addition, four patients with relapsed glioma had evaluable recurrent tumor tissues. We found that the paired closest TISF cf-tDNA sample at recurrence contained all the mutations detected in the recurrent tissue, while the matched initial and recurrent tissues only shared a portion of mutations (**Figures 2B–E**). More interestingly, two other patients were diagnosed with pseudoprogression by a second surgery, and their closest preoperative cf-tDNA samples were mutation-negative or remained stable from previous tests (**Figures 2F, G**).

Together, the dynamic consistency between tumor tissue and cf-tDNA samples indicated that the representativeness of initial tumors for the glioma decreased with tumor evolution, and the real-time genomic status could be complemented by the TISF cf-tDNA.

### Serial cf-tDNA depicts the evolutionary patterns and trajectories of glioma genomes

**Figure 3:**
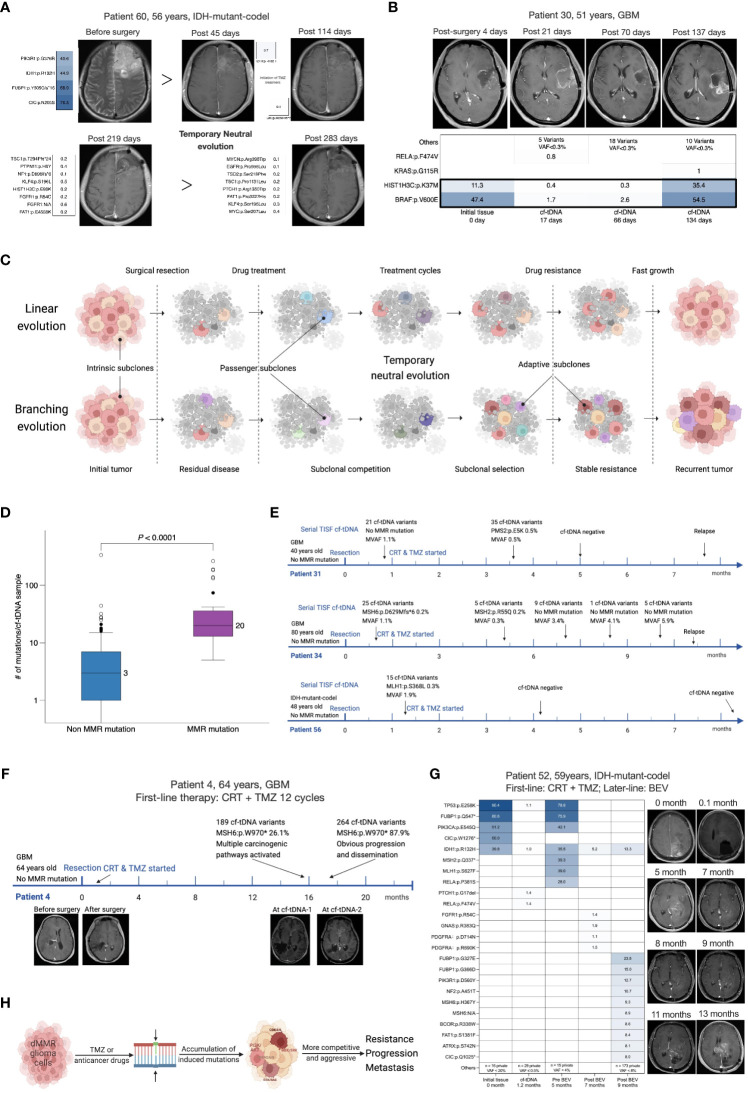
Serial cf-tDNA reveals the evolutionary trajectories and resistant mechanisms of glioma. **(A)** Illustrative example for serial cf-tDNA identifying a period of neutral evolution during treatment. **(B)** Serial cf-tDNA depicts the evolutionary trajectories of glioma at multiple timepoints. **(C)** Illustrative diagram of tumor evolution during the treatment resistance revealed by serial cf-tDNA profiling. **(D)** Number of mutations in cf-tDNA samples with or without MMR mutations. **(E)** In the three patients, MMR mutations disappeared later after their emergence. **(F, G)** MMR mutations caused hypermutation and infiltrative progression in the two patients with recurrent gliomas. **(H)** Schematic diagram of MMR deficiency-induced hypermutation and progression. cf-tDNA, cell-free tumor DNA; MMR, mismatch repair.

Next-generation sequencing studies have captured a variety of evolutionary patterns of cancer shaping and development, mainly including linear evolution (LE), branching evolution (BE), and neutral evolution (NE) (1, 2, 17–19). Combining serial cf-tDNA samples with tumor tissues could identify dynamic evolutionary patterns of the glioma (**Figure 3C**). For example, the TC cf-tDNA samples in the majority at recurrence suggested that new adaptive mutations emerged in the offspring tumor cells, which was consistent with a BE pattern. One illustrative example was again with patient 67, a 64-year-old man with IDH-mutant-noncodel glioma. Both his relapsed cf-tDNA sample and recurrent tumor tissue were detected to have newly emerging CDKN2A:p.L16Pfs*9 and PDGFRA:p.V536E mutations compared to the initial tissue (**Figure 2B**), indicating that the relapsed tumor had evolved more malignant branches featured with CDKN2A loss (20). In addition, during radio- and chemotherapy, many instant low-VAF passenger mutations appeared and disappeared in serial cf-tDNA samples, which might reflect a temporary period of neutral evolution of the glioma genome under therapeutic pressure. For example, patient 60 was a 56-year-old man with IDH-mutant-codel glioma. His first TISF cf-tDNA sample after surgery was detected to have a tissue-shared IDH1:p.R132H mutation, while in the subsequent three cf-tDNA samples spanning 7.0 months during the TMZ chemotherapy, one, eight, and eight low-VAF mutations were found, with none of them shared, and they remained stable meantime as detected by MRI (**Figure 3A**). Relapsed gliomas having BC cf-tDNA samples might experience an LE pattern, where the most significant selection pressure shaping the tumor genome happened before the diagnosis of glioma, while the following treatment only exerted a subtle effect on the relapsed genomic structure. In addition, analysis of the sequence of the emergence of the driver mutations in the serial cf-tDNA can trace the subclonal evolutionary trajectories of glioma. For example, serial cf-tDNA analysis revealed the patient 30’s GBM was linearly driven and progressed by successive appearance of subclones featured with BRAF:p.V600E and HIST1H3B:p.K37M mutations (**Figure 3B**).

### Selection of pre-existing MMR-deficient tumor cells as a mechanism of resistance and infiltrative progression

Mismatch repair (MMR) deficiency and hypermutation in the glioma genome were reported as related to the TMZ-resistant mechanisms (21). In 107 patients’ initial tumor tissues, we only detected MMR mutations (MSH6, MLH1, MSH2, or PMS2) in two IDH-mutant initial tumors (2 of 107, 1.8%) (**Figure 1B**), while MMR mutations were present in 7/43 (16.3%) patients’ temporally paired cf-tDNA samples; none of their tissues had MMR mutations identified (**Figure 1D**). Of 107 patients, 25 (23.4%) had at least one cf-tDNA sample identified as MMR mutations (**Figures 1A, B**). A total of 35 cf-tDNA samples with MMR mutations had far more mutations than those 170 without (median 20 vs 3 mutations/sample, *p* < 0.0001, Mann–Whitney test) (**Figure 3D**). Of note, not all MMR mutations would persist after its emergence and cause hypermutation. For example, patient 72 with IDH-mutant-noncodel glioma had a cf-tDNA sample with two transient MMR mutations (VAF 0.1%) during the TMZ treatment, but the two mutations disappeared in the following cf-tDNA samples and did not cause many mutations. Low-VAF MMR mutations were found in early cf-tDNA samples of patients 34 and 56 and then vanished with treatment (**Figure 3E**). However, relapsed cf-tDNA samples with high-VAF MMR mutations were prone to a high number of mutations and to tumor dissemination or distant recurrence. For example, multiple MMR mutations (the highest VAFs were 26.1% and 87.9%) were detected in the 1-month-apart two relapsed cf-tDNA samples of patient 4 with GBM, with 189 and 267 mutations identified in the two samples; meanwhile, the tumor spread rapidly to other regions (**Figure 3F**). Patient 52 with IDH-mutant-codel glioma received BEV treatment after tumor recurrence, the first post-treatment cf-tDNA sample at 2 months showed molecular remission, while the second cf-tDNA sample at 4 months was detected to have MMR mutations (highest VAF 9.3%) with 184 somatic mutations, and the tumor disseminated toward other regions as well (**Figure 3G**).

These phenomena supported the hypothesis that MMR-deficient cells are pre-existing in the tumor and that TMZ treatment exerts certain selection pressure biased toward MMR-deficient cells (21). However, the direct survival advantage of MMR-deficient cells may derive from that treatment-induced numerous mutations are more likely to activate a variety of carcinogenic signaling pathways, making the subclones more resistant and invasive (**Figure 3H**).

### Prognostic and relapse diagnostic value of serial cf-tDNA assessment

**Figure 4:**
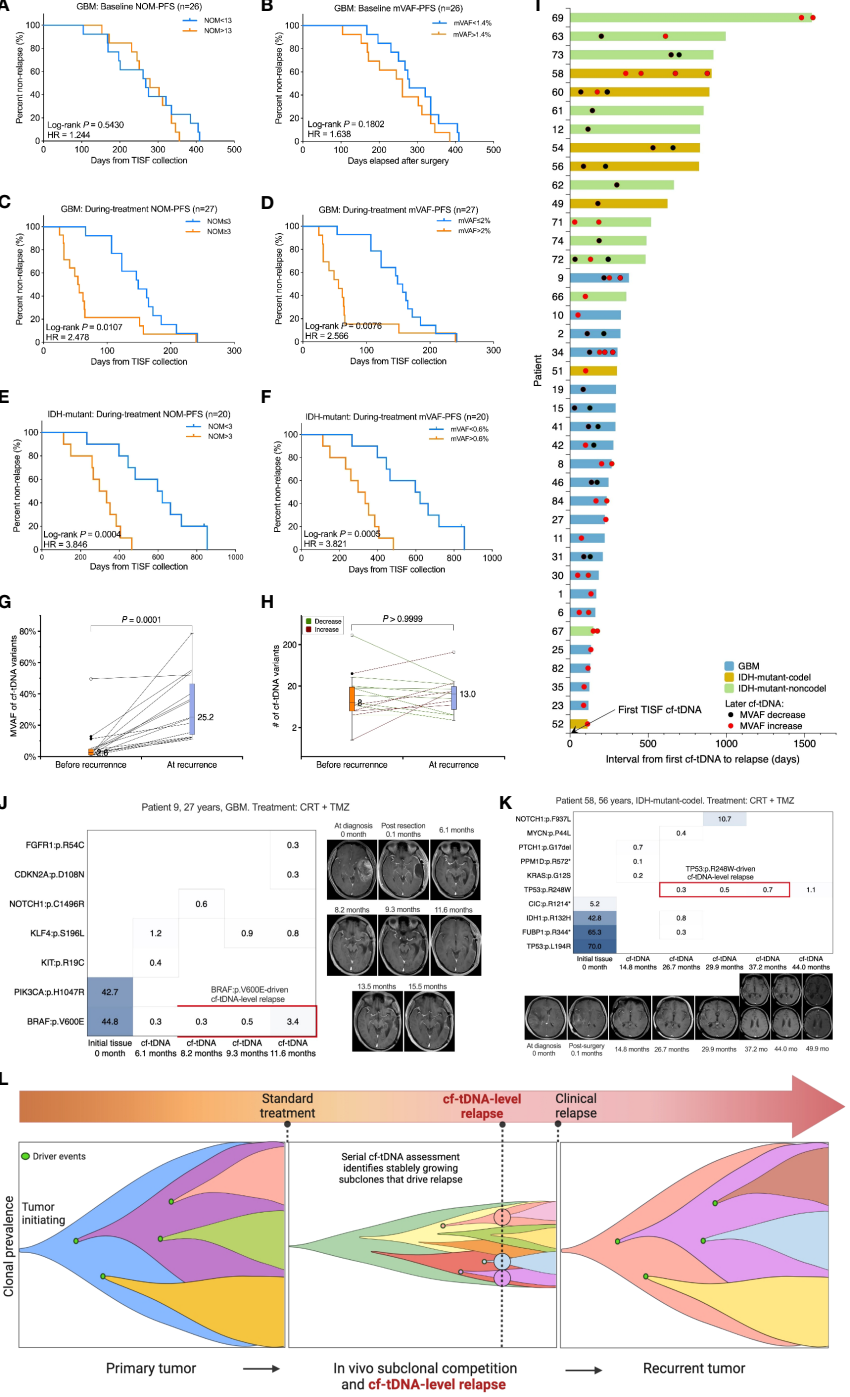
Prognostic and relapse diagnostic value of serial cf-tDNA assessment. **(A, B)** The number of mutations (NOM) or the maximal VAF (mVAF) in the baseline cf-tDNA samples (before antitumor treatment) was not predictive of recurrence. Medians as cutoffs. Log-rank test. **(C–F)** A larger NOM or a higher mVAF during treatment was associated with a shorter progression-free survival (PFS) following TISF collection in both IDH-wild-type and IDH-mutant gliomas. Medians as cutoffs. Log-rank test. **(G)** The mVAFs of relapsed cf-tDNA samples were higher than those before recurrence. Wilcoxon matched-pairs signed rank test. **(H)** NOMs did not increase at recurrence. Wilcoxon matched-pairs signed rank test. **(I)** Serial cf-tDNA mVAF monitoring during tumor recurrence in 39 evaluable patients. **(J, K)** Two illustrative examples for serial cf-tDNA identifying progression-driving mutations before imaging recurrence. **(L)** Diagram of tumor resistance evolution and serial cf-tDNA diagnostic cf-tDNA-level relapse in gliomas. VAF, variant allele fraction; TISF, tumor *in situ* fluid.

The NOM or the mVAF in the baseline cf-tDNA samples (before antitumor treatment) was not prognostic for progression in evaluable GBM patients (**Figures 4A, B**). However, a larger NOM or a higher mVAF in the during-treatment cf-tDNA sample was associated with shorter PFS following TISF collection in both IDH-wild-type (NOM: hazard ratio 2.478, log-rank *p* = 0.0107; mVAF: hazard ratio 2.566, log-rank *p* = 0.0076) and IDH-mutant gliomas (NOM: hazard ratio 3.846, log-rank *p* = 0.0004; mVAF: hazard ratio 3.821, log-rank *p* = 0.0005) (**Figures 4C–F**). Most tumors whose mVAF has continuously increased progressed shortly (**Figure 4I**). The mVAFs of relapsed cf-tDNA were significantly higher than those before recurrence (median mVAF 25.2% vs 2.6%, *p* = 0.0001, Wilcoxon matched-pairs signed rank test) (**Figure 4G**), while the NOM did not increase at recurrence (**Figure 4H**).

The high sensitivity of TISF cf-tDNA in glioma patients may allow continuous molecular-level monitoring of the tumor evolution from a minuscular burden, making it possible to identify responsible genomic alterations that drive tumor progression and recurrence at the earliest possible time. For example, patient 9 was a 27-year-old man with GBM. During the TMZ treatment, a BRAF:p.V600E mutations were detected in three serial cf-tDNA samples spanning 3.4 months, and the VAF of the variant increased from 0.3% to 3.4%, while the contemporaneous MRI findings were negative until slight marginal progression appeared on the MRI 4 months later (**Figure 4J**). Similarly, patient 58, a 56-year-old woman with IDH-mutant-codel tumor, had a TP53:p.R248W mutation with gradually increasing VAF (from 0.3% to 0.7%) detected in three sequential cf-tDNA samples over 10.5 months, and the MRI changes were observed until 6.8 months later (**Figure 4K**).

In the two examples, before imaging changes, serial cf-tDNA identified BRAF:p.V600E and TP53:p.R248W mutations that were responsible for driving the two tumors’ progression. We called that cf-tDNA-level relapse because the serial detection and increase indicated that the tumor had developed stable resistant subclones that began to dominate the fast growth of the relapse tumor, which could be detected by imaging modalities after a subsequent period of volume expansion (**Figure 4L**).

### Serial cf-tDNA profiling reveals many potentially actionable genomic alterations during glioma evolution

The dynamic heterogeneity of glioma genomes cannot be fully characterized by sequencing of resected tumor tissues, thereby hindering the temporal precision medicine during the tumor evolution for the affected patients. To evaluate the potential therapeutic utility of serial TISF cf-tDNA analysis, we assessed the number of cf-tDNA variants that could potentially be clinically targeted by available drugs. We estimated 223 different cf-tDNA variants are potentially clinically targetable, of which 174 (78.0%) were not found in any patient’s tumor tissues (**Supplementary Table 4**). These cf-tDNA-unique variants are targetable primarily with small molecules that inhibit PI3K/AKT/mTOR, RTK/RAS, or P53 signaling pathways (**Supplementary Figure 1**), all of which have been approved in other cancers and are being tested clinically in gliomas.

## Discussion

In the current study, 87.3% of cf-tDNA samples harbored tumor-derived mutations. The high sensitivity surpassed previous studies based on CSF or blood sequencing, which corroborated that sampling closest to the tumor increases sensitivity (10). In the cohort, TISF cf-tDNA was positive for some patients despite that their tumors were totally resected on MRI, while some other patients were detected to be negative after the initiation of postoperative treatment. We conjecture that the volume threshold for TISF cf-tDNA detection may exceed the MRI assessment, and further evaluation of tumor metabolic modalities may be needed to identify the relationship between tumor load and cf-tDNA detectivity. In the study, the genomic landscapes of temporally paired cf-tDNA samples were not completely consistent with those of tumors, indicating that there is a wide range of subclones in gliomas that are not fully covered by tissue sequencing. Subsequent consistency dynamics analysis further weakened the representativeness of the initial tumor for the ever-evolving glioma. On the contrary, the comparison between recurrent tumors and matched cf-tDNA samples evidenced that cf-tDNA can complement the real-time genome of gliomas as well as distinguish pseudo-progression. In short, these observations reinforce the necessity of repeated biopsies for gliomas, which can be complemented by serial TISF cf-tDNA. In addition, considering the dissemination and distant relapse of some gliomas, it may be necessary to integrate TISF with CSF as well as blood to decode the mechanism of intracranial dissemination and extracranial metastasis of gliomas.

Researchers represented by the Glioma Longitudinal AnalySiS (GLASS) consortium (22) are working on delineating the comprehensive multiomic evolutionary landscape of recurrent gliomas, providing new bases for new pharmacology and therapies (1, 20). Serial cf-tDNA analysis allows multi-timepoint characterization of the evolutionary trajectory of tumors. In the study, we found that glioma experienced a period of NE during treatment, during which the tumor remained at a low-load status, suggesting that the treatment was effective during this period. At tumor recurrence, there were two different evolutionary results, BE and LE. The two patterns may inform two sources of resistant subclones in recurrent gliomas, intrinsic and adaptive resistance. In the BE, the resistance of recurrent gliomas consists of both, while in the BE, it mainly includes the intrinsic resistance that was formed before the diagnosis. Serial analysis of the emerging sequence and changing abundance of genomic alterations in cf-tDNA samples can depict the continuous genomic trajectories of the resistant evolution and offer more timing options for the application of new therapies.

Hypermutations are rare (only 2%) in newly diagnosed gliomas, while in recurrent tumors, the proportion rises to 16.6% (21). Existing studies have demonstrated that hypermutation not only cannot render a survival benefit from immunotherapy for patients (21) but also can lead to an increasing number of stem-like neoplastic cells in recurrent tumors, thereby reducing the overall survival of patients (20). Touat et al. (21) showed that exposure of MMR-deficient tumor cells to TMZ causes hypermutation; however, this is not enough to fully explain the great difference in the proportion of hypermutation between initial and recurrent tumors. In our study, we found that 1.8% of initial tumors harbored MMR mutations, while if taking the cf-tDNA residual disease into account, the proportion would rise to 20% (9/45). In addition, we observed that MMR mutations in some patients may disappear with treatment. These findings suggest that MMR-deficient subclones pre-exist in some gliomas before TMZ treatment, while TMZ is not the cause of MMR deficiency, and the selection pressure for MMR-deficient cells is not very strong as well; otherwise, the MMR-deficient subclones would not disappear once they emerged and would expand rapidly to dominate the tumor’s clonal structure. We hypothesize that the determinant of the development of MMR-deficient cells into hypermutated recurrent gliomas is that the large number of passenger mutations caused by TMZ plus MMR deficiency successfully activate a variety of carcinogenic pathways, rendering these subclones more resistant and invasive. This means that recurrent gliomas with hypermutation are more malignant and more prone to infiltrative metastasis. Our findings support the development and application of liquid biopsy panels that specifically detect MMR genes to monitor the TMZ treatment in order to cease or change the treatment at appropriate timings.

In the study, we showed that the cf-tDNA parameters (NOM and mVAF) especially the serial cf-tDNA assessment during the adjuvant treatment were predictive of progression, offering physicians new opportunities to identify poor responders for whom more intensified or experimental therapy can be implemented. On the contrary, early cf-tDNA negativity or serial cf-tDNA keeping stable after the initiation of treatment could be evaluated as an additional criterion for treatment de-escalation in patients who are otherwise considered to have low-risk progressive disease. The lack of an association between baseline cf-tDNA parameters and progression is expected, as the baseline signal only represents residual disease detectable by cf-tDNA sequencing, which could be effectively eradicated by subsequent adjuvant treatment. More importantly, serial cf-tDNA profiling can identify the gradual increase in the VAF of specific driver mutations, enabling the early molecular diagnosis of glioma recurrence before imaging changes, making it possible for the second-line therapy to be initiated at a lower tumor burden. These findings support serial TISF cf-tDNA profiling as a promising complementary tool to risk stratification of gliomas defined at diagnosis, which warrants integration into future glioma clinical trials.

Targeted therapy is a promising treatment regimen to cure gliomas (23). It has been known that multiple driver events and signaling pathways are responsible for the formation, resistance, and progression of glioma, which may allow patients to benefit from various targeted therapies during tumor evolution. In the current study, we assessed that 78% of cf-tDNA variants that could potentially be clinically targeted by available drugs were not found in any patient’s tumor tissues. This indicates that there are many potential therapeutic vulnerabilities during the tumor evolution that are not identified in the initial tumor tissues. In the future, real-time characterization of potential therapeutic targets by serial cf-tDNA is needed to improve precision oncology clinical trials in gliomas.

## Conclusions

In conclusion, the results of this study support the recommendation of integrating serial TISF cf-tDNA profiling into clinical practice and trials for patients with glioma to provide real-time data on genomic evolution and the development of resistance to identify potentially clinically targetable mutations, bringing precision medicine closer to patients with glioma.

## Data availability statement

The datasets presented in this study can be found in online repositories. The accession link is https://figshare.com/articles/dataset/Data_for_10_3389_fonc_2023_1238607/24186477.

## Ethics statement

The studies involving humans were approved by Henan Provincial People’s Hospital Ethical Committees. The studies were conducted in accordance with the local legislation and institutional requirements. The participants provided their written informed consent to participate in this study.

## Author contributions

Conception and design: XB, ZS, AZ. Provision of study materials or patients: ZS, CB, JM, SX, ZZ, GG, YG, and CB. Collection and assembly of data: ZS, CB, JM, SX, ZZ, GG, and YG. Data analysis and interpretation: ZS, AZ, XB, CB, and ZC. Manuscript writing: ZS wrote the first draft, and all authors revised it. All authors contributed to the article and approved the submitted version.
