# Supplementary material for: Tracking tumor evolution during the first-line treatment in brain glioma via serial profiling of cell-free tumor DNA from tumor in situ fluid
Source: Front Oncol. 2023 Oct 17;13:1238607. doi: 10.3389/fonc.2023.1238607 (PMC10619896; doi:10.3389/fonc.2023.1238607)
Supplement: Supplementary file 1 [file Image_1.pdf]

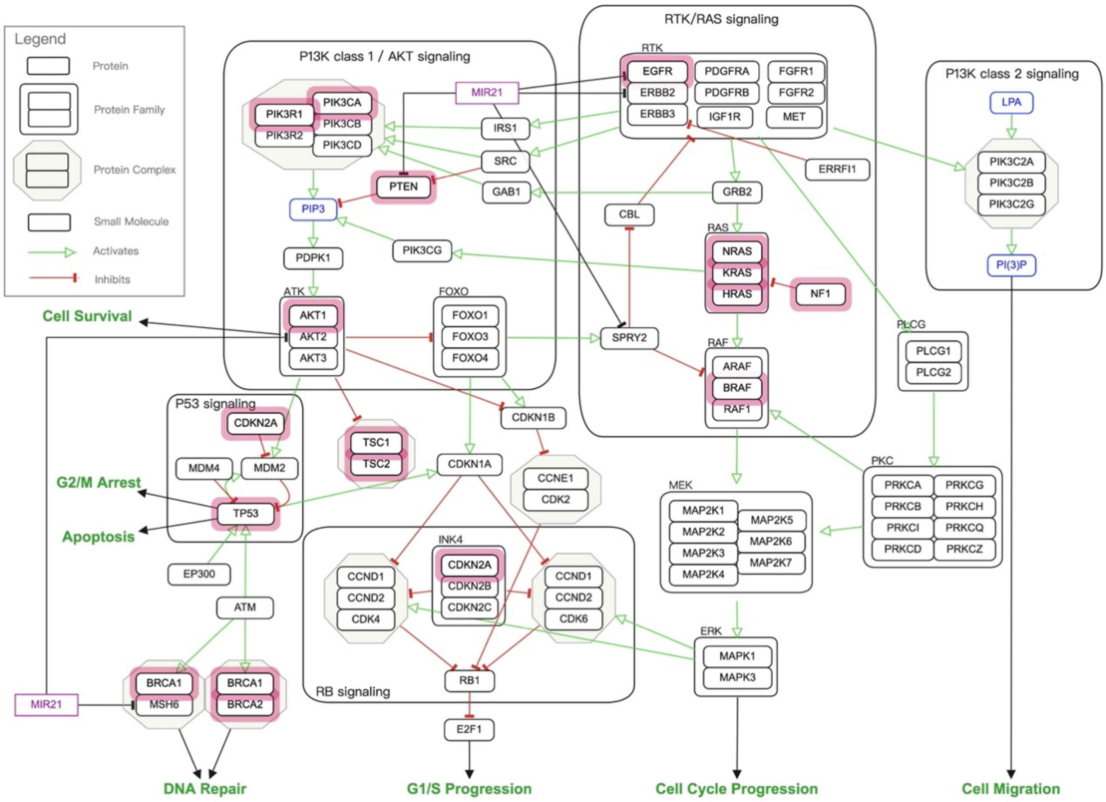

Figure S1. Potentially actionable cf-tDNA mutations in GBM pathways. The integrative pathways were cited from the WikiPathways (https://www.wikipathways.org/index.php?title=Pathway:WP2261&oldid=123065), genes detected in the cf-tDNA mutations are marked with red frames.
